# Automated Messaging Delivered Alongside Behavioral Treatment for Weight Loss: Qualitative Study

**DOI:** 10.2196/50872

**Published:** 2023-11-06

**Authors:** Michael Berry, Lauren Taylor, Zhuoran Huang, Christina Chwyl, Stephanie Kerrigan, Evan Forman

**Affiliations:** 1 Department of Psychological and Brain Sciences Drexel University Philadelphia, PA United States; 2 Center for Weight, Eating and Lifestyle Science Drexel University Philadelphia, PA United States; 3 Stephanie Kerrigan, LLC New Haven, CT United States

**Keywords:** mobile health technology, weight loss, tailored messaging, lifestyle modification, mobile health, mHealth, messaging, weight loss, intervention, overweight, obesity, qualitative, thematic analysis

## Abstract

**Background:**

Mobile health interventions for weight loss frequently use automated messaging. However, this intervention modality appears to have limited weight loss efficacy. Furthermore, data on users’ subjective experiences while receiving automated messaging–based interventions for weight loss are scarce, especially for more advanced messaging systems providing users with individually tailored, data-informed feedback.

**Objective:**

The purpose of this study was to characterize the experiences of individuals with overweight or obesity who received automated messages for 6-12 months as part of a behavioral weight loss trial.

**Methods:**

Participants (n=40) provided Likert-scale ratings of messaging acceptability and completed a structured qualitative interview (n=39) focused on their experiences with the messaging system and generating suggestions for improvement. Interview data were analyzed using thematic analysis.

**Results:**

Participants found the messages most useful for summarizing goal progress and least useful for suggesting new behavioral strategies. Overall message acceptability was moderate (2.67 out of 5). From the interviews, 2 meta-themes emerged. Participants indicated that although the messages provided useful reminders of intervention goals and skills, they did not adequately capture their lived experiences while losing weight.

**Conclusions:**

Many participants found the automated messages insufficiently tailored to their personal weight loss experiences. Future studies should explore alternative methods for message tailoring (eg, allowing for a higher degree of participant input and interactivity) that may boost treatment engagement and efficacy.

**Trial Registration:**

ClinicalTrials.gov NCT05231824; https://clinicaltrials.gov/study/NCT05231824

## Introduction

Approximately 40% of adults worldwide [1] and 70% of adults living within the United States [2] are overweight or obese, which is associated with significantly higher mortality and morbidity risks (eg, cardiovascular disease, cancer, and type 2 diabetes) [3]. Even losses of 5% or more of baseline weight, achievable through intensive behavioral weight loss treatment (typically involving 14 or more group or individual counseling sessions over 6-12 months [4]), are associated with health improvements [5,6].

Limited access to intensive weight loss treatment due to clinician scarcity and cost [7,8] has led to an interest in scalable low-cost mobile health (mHealth) interventions, in which intervention materials are often relayed through text messages or mobile apps [9,10]. Many mHealth weight loss interventions use *automated messaging*, which offers computerized support encompassing education, motivation (eg, encouraging feedback messages), and data monitoring [11]. Many automated messaging systems described in the literature have been fairly simplistic, for example, providing generic weight loss tips or reminders (eg, to adhere to a regular eating schedule) [12,13]. Other systems have been more sophisticated, for example, providing detailed data summaries (eg, progress toward physical activity and dietary goals) [14,15], messaging content tailored to user characteristics or preferences [16,17], or targeted strategy suggestions informed by momentary risk factors for dietary nonadherence (eg, increased stress) [16,17]. Notably, among the fully automated weight loss trials included in a recent systematic review from our research group [18], most automated messaging systems for weight loss were relatively simplistic, with only 27 of 44 using individually tailored messaging and only 11 providing users with feedback on data patterns.

Meta-analytic reviews suggest that messaging-based interventions alone have limited effectiveness for significant weight loss (only 1%-2% compared to minimal intervention) [10,19]. One approach to improve the effectiveness of automated messaging is to evaluate increasingly technologically advanced systems, such as artificial intelligence (AI)–based chatbots that mimic human counselors [20]. However, increased complexity alone does not guarantee better outcomes. For example, one trial of a sophisticated conversational chatbot reported similar weight loss results to simpler messaging systems (n=70; 2.38%) [21]. An alternative strategy involves gathering user input to identify feasible, acceptable, and potentially effective messaging components for weight loss [22], before conducting additional clinical trials of fully developed novel messaging systems. A few prior studies applying this user-centered feedback approach have underscored the importance of message tailoring, reinforcement of weight loss skills, and supportive accountability [12,23-25]. However, these studies all evaluated relatively simplistic messaging systems that lacked advanced individual tailoring or feedback (eg, on adherence to calorie targets). User experience data from more advanced messaging systems that include such features [12,23-25] is limited, hindering the refinement of automated messaging as an intervention strategy.

As such, this study evaluated user experiences with a novel, advanced automated messaging system for weight loss, which was developed as part of an ongoing behavioral weight loss trial, Project ReLearn [26]. Similar to other advanced automated messaging systems [12,23-25], the Project ReLearn system provided detailed, highly tailored feedback on individuals’ dietary data patterns (eg, weight and dietary self-monitoring) and suggested targeted behavioral strategies based on these patterns. An additional unique feature of the Project ReLearn system was the ability to vary messaging tone and content (eg, praise vs suggestions for improvement) over time. The goal of this feature was to further enhance message tailoring [27], limit message repetitiveness, and maintain user engagement, given the known challenge of retaining engagement within digitally delivered behavioral interventions [28]. Furthermore, unlike in prior studies, Project ReLearn combined automated messages with human counseling, which is known to enhance digital weight loss intervention efficacy [29,30]. This study design also allowed participants to directly compare the automated messaging system with their interactions with human coaches.

The central aim of this study was to understand the experiences of participants with the automated messaging system, both positive and negative, and generate ideas for improvement through structured qualitative interviews and thematic analysis. We were not only interested in identifying what participants found helpful about the system but also anticipated that participants would identify system weaknesses (eg, lack of conversational ability) to inform the development of more effective automated messaging systems for weight loss. Additionally, to provide context for the qualitative findings, this report details the design of the messaging system and presents participants’ ratings of message acceptability on various domains related to the rationale for the system (eg, to help users make the changes to their behavior required to lose weight).

## Methods

### Ethical Considerations

This study was conducted as part of an ongoing clinical trial that received ethical approval from the Drexel University institutional review board (Protocol #2102008368). Written informed consent was obtained from all participants. All study data were deidentified prior to analysis and paper preparation. Identifying participant information collected through the parent trial was secured in a password-protected encrypted database accessible to study staff. Participants were compensated US $75 for completing the 6-month assessment and US $100 for completing the 12-month assessment.

### Parent Trial

The automated messaging system described in this study is currently being deployed as part of an ongoing behavioral weight loss trial, Project ReLearn (ClinicalTrials.gov: NCT05231824). In this trial, individuals with overweight or obesity living within the United States are randomized to receive either 1 year of standard behavioral weight loss groups (BWL-S) or AI-optimized behavioral weight loss (BWL-AI) delivered remotely via videoconference. Participants in both groups receive 1 month of weekly group sessions (phase 1). Participants in BWL-AI receive 1 month of weekly group sessions, followed by 11 months where they receive 1 of 3 possible interventions each week: a small videoconference group with a master’s degree–level counselor, a 12-minute individual video call with the master’s degree–level counselor or paraprofessional, or an automated message (“coaching message”). Within the BWL-AI group, weekly intervention assignment is fully automated by an algorithm that uses the AI technique of reinforcement learning [31,32]. In brief, this algorithm continuously monitors and models participants’ digital data (eg, weight and physical activity) to predict the intervention assignment most likely to maximize the collective amount of weight loss across the BWL-AI condition in a given week. These predictions are then used to select an intervention for each participant each week, within the constraints of counselor time and required group size (n=8). Of note, AI is used only for the purpose of intervention selection in Project ReLearn and does not generate automated messages or other intervention content. As such, participants in the BWL-AI group receive automated messages instead of human counseling (eg, group and individual calls) during weeks where they are assigned to receive an automated message and never receive messages and human counseling during the same week.

**Figure 1:**
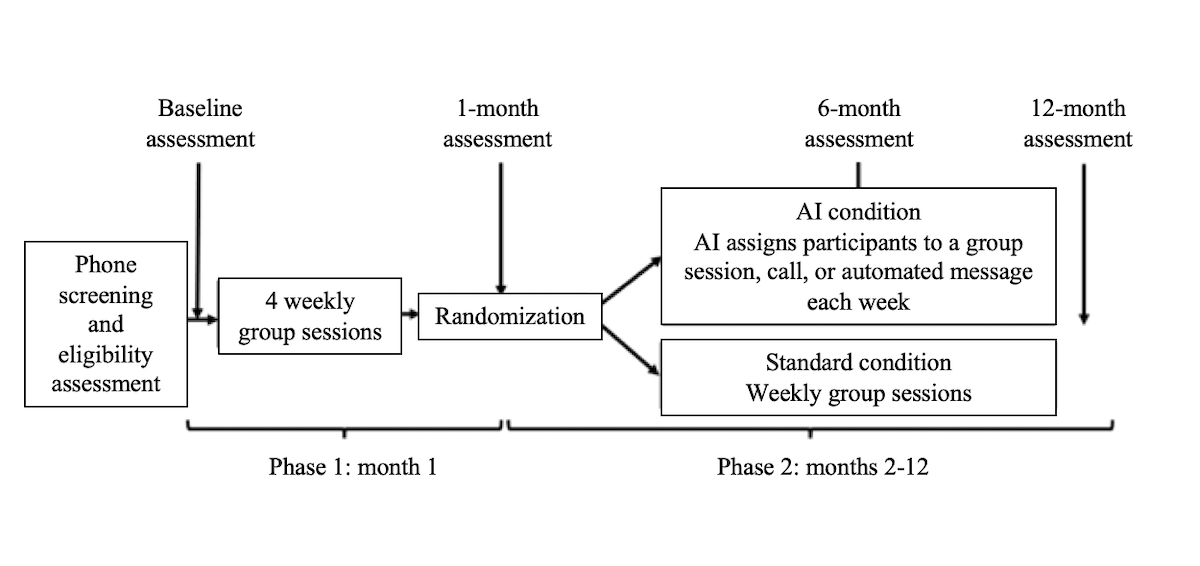
Design overview for the parent trial, Project ReLearn. AI: artificial intelligence.

All participants in Project ReLearn adhere to weekly physical activity goals and a target “calorie range” (defined as the range between the participant’s selected calorie goal and 300 calories below the goal). Participants collaboratively establish these goals with their counselors during the first month of the program (ie, phase 1) and can request to adjust their goals during phase 2. All participants are loaned a wrist-worn physical activity tracker (Fitbit Inspire 2) and are instructed to self-weigh daily using a digital body weight scale (Fitbit Aria Air) and record all dietary intake using the Fitbit mobile app. BWL-AI participants additionally use a study-specific mobile app (“ReLearn app”) to access program materials (eg, written informational modules). An overview of the parent trial design, including the timing of assessments and various intervention components, is presented in Figure 1. Additional details about the design, rationale, and eligibility criteria for the parent trial are reported in a previously published protocol paper [26].

### Participants

Participants in Project ReLearn are adults who are overweight or obese (aged 18-70 years; BMI 27-50 kg/m^2^). This report includes data obtained from the 40 BWL-AI participants active in treatment at the time data for this project was obtained between January and March 2023. Eight additional BWL-AI participants who withdrew from treatment prior to data collection for this report are not included. Participants were surveyed either at the midtreatment (6 months from baseline, n=20) or posttreatment assessment (12 months from baseline, n=20).

### Design of the Automated Messaging System

#### Variables Referenced by the Coaching Messages

Coaching messages were constructed using the following data variables, calculated by a custom web portal using raw data obtained from each participant’s Fitbit account weekly: (1) raw *weight change* over the past week (lbs); (2) cumulative *percent weight change* since the first week of the program; (3) *number of days self-weighed* during the past week; (4) minutes of moderate to vigorous activity (MVPA *minutes*) during the past week, defined as the total number of “active zone minutes” logged in Fitbit for the week (determined based on the participant’s estimated current and resting heart rate at each minute throughout the week); (5) *number of activity tracking days* defined based on whether the participant recorded any MVPA minutes in Fitbit each day; (6) *average calorie intake* over the past week; (7) *number of days within, above and below target calorie range* during the past week; and (8) *app use* (whether the participant opened the ReLearn app during the past week). Weekly weight change was further categorized as either weight loss (<–0.4 lbs), weight maintenance (–0.4 lbs to 0.4 lbs), or weight gain (>0.4 lbs). Overall adherence to dietary self-monitoring was inferred from the number of days within or above the calorie range each week (days below the calorie range were considered likely evidence of underreporting calorie intake).

#### Coaching Messages

##### General Summary

**Figure 2:**
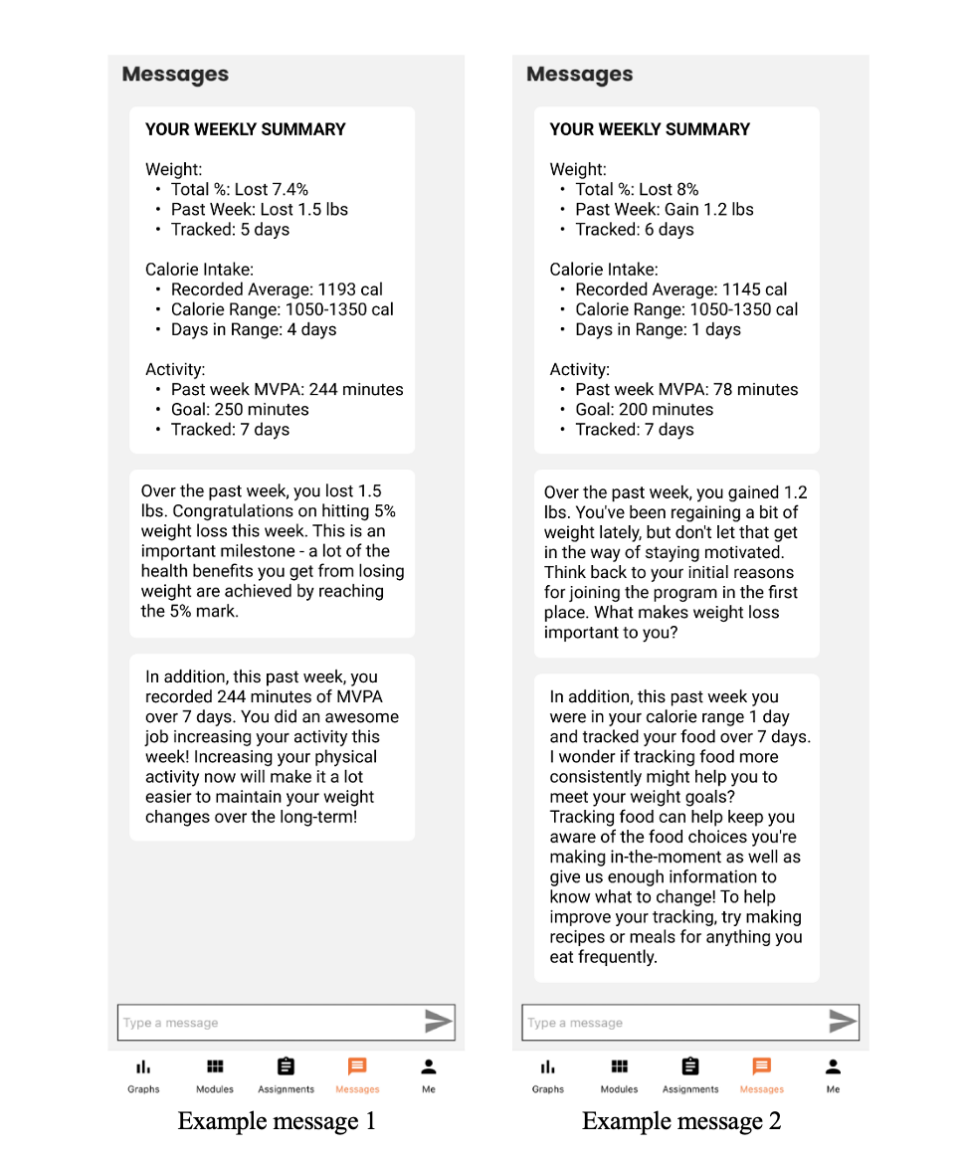
Two examples of coaching messages. MVPA: moderate to vigorous activity.

Figure 2 depicts 2 example messages. Coaching messages contained three messaging components: (1) a “summary box” of weight and behavioral data patterns during the past week, (2) tailored feedback on recent weight change patterns, and (3) tailored feedback on recent adherence to behavioral (eg, dietary and physical activity) goals. Coaching messages were delivered through the “messages” tab of the ReLearn app at noon on the participant’s scheduled intervention day. Message content was developed collaboratively by our research team, which was comprised of clinical psychology research faculty, postdoctoral fellows, and PhD students. All members of this group have substantive experience with lifestyle modification treatment, mHealth interventions, and evidence-based principles of behavior change.

##### Component 1: Summary Box

The summary box component succinctly presented participants’ progress toward their daily or weekly weight, calorie intake, self-monitoring, and physical activity goals. Participants were informed of their cumulative percent weight change from baseline and whether they lost, gained, or maintained weight in the past week. Participants received an estimate of their average daily calorie intake during the past week, a reminder of their target calorie range, and the number of days that they were within this range during the past week. Participants were also informed of their estimated total MVPA minutes from the past week, number of activity tracking days, and current physical activity goal.

##### Component 2: Weight Feedback

The purpose of the weight feedback messaging component was to provide tailored feedback on weight change during the past week and prompt participants to consider what might be helping or hindering their overall progress with weight loss. This messaging component was selected from a pregenerated messaging bank using participant-specific data criteria (past-week weight loss, weight loss during the 3 weeks prior to last week, cumulative percent weight loss, and whether the participant reports currently trying to lose or maintain weight). For example, a participant pursuing weight loss who has overall gained weight since starting the program experienced a weight gain during the past week and experienced a mix of gains and losses (overall gain) during the prior 3 weeks would receive the following message:

It seems like you are having some trouble getting on a consistent weight loss trajectory. Think back to those weeks when you lost weight and whether there was anything you did differently from usual those weeks. Consider making a list of concrete things you need to do to get back on track.

To limit message habituation, participants who met the same exact data criteria more than once during the program received a different message each time (ie, more than 1 message can be selected based on a given set of criteria). Participants additionally received a brief positive reinforcement message upon reaching significant weight loss milestones (5% and 10% weight loss) for the first time. Participants who did not self-weigh during the past week did not receive any weight feedback and instead received a written reminder to regularly self-weigh if they would like to continue experiencing benefits from the program.

##### Component 3: Behavioral Feedback

The purpose of the behavioral feedback messaging component was to provide tailored feedback on adherence to behavioral prescriptions (dietary self-monitoring, reduced calorie intake, and increased physical activity). Similar to the weight feedback component, the behavioral feedback component was selected from a pregenerated messaging bank based on participant-specific data criteria. Unlike the weight feedback component, the behavioral feedback component contained 2 distinct subcomponents and used an additional selection criterion known as a “theme.” The component was constructed in a multistage process as follows: (1) selection of a “theme”; (2) selection of a “parent” subcomponent corresponding to the theme; and in some cases, (3) selection of a “targeted feedback” subcomponent, which provided either reinforcement for goal progress or a strategy suggestion to improve progress. Themes determined the overall tone of the behavioral feedback component (eg, praise vs problem-solving) and aligned the weight feedback component with the behavioral feedback component. For example, some themes were more likely to be selected for participants who recently experienced difficulty losing weight (eg, the “Goal Declining” theme), whereas others for participants who recently experienced more consistent weight loss (eg, the “Goal Improving” theme). Themes were selected probabilistically based on participants’ current level of weight loss progress (see Table S1 in Multimedia Appendix 1, “Overview of behavioral feedback message themes,” for an example) to further reduce repetitiveness. In addition, the same theme could not be selected in consecutive weeks for a given participant.

After a theme was selected, the “parent” subcomponent was then identified from a separate message bank based on specific data criteria, which could be related to weight (eg, lost weight during the past week; of note, the behavioral message component itself never included any feedback on weight) and behavior-related (eg, self-weighed on fewer than 5 days during the past week). For brevity, each parent subcomponent only addressed a single behavioral prescription. If a participant met the criteria for multiple “parent” subcomponents, then a subcomponent was randomly selected (of note, the parent subcomponent also cannot provide feedback on the same behavioral prescription 2 weeks in a row, to further limit repetitiveness). To ensure that participants always met the criteria for at least 1 parent subcomponent, certain subcomponents have criteria that almost all participants would meet (eg, opening the ReLearn app once during the past week). Finally, the end of each parent subcomponent included a placeholder field specifying whether a targeted feedback subcomponent should also be selected (not all parent subcomponents include this field). The targeted feedback subcomponent either provided reinforcement for progress toward goals (eg, “Great job staying so consistent with self-weighing!”) or suggested a strategy to improve adherence (“Try setting a reminder on your phone, tablet, or computer to weigh in each morning”). The targeted feedback subcomponent was selected randomly from its own, separate message bank and matched to the specific domain (eg, increasing physical activity) of the parent subcomponent.

##### Rescue Mode

To accommodate situations where participants temporarily wish to pause their weight loss efforts, participants were given an option within the ReLearn app to enable “rescue mode.” When activated, this feature entirely disabled the coaching messages and replaced them with an encouraging “check-in” message that did not reference the participant’s level of progress (eg, “It's been a few weeks, so we wanted to check in—are there any goals you'd like to work towards this week? If so, just click here and you can change your settings! If not, that's okay, just keep taking care of yourself and we'll continue to check in!”). Of note, no participants had yet activated rescue mode by the time of their assessment for this study.

##### Coaching Message Evaluation

###### Acceptability Measures

To evaluate the acceptability of the messaging system and provide additional context for the qualitative assessment (described in the next section), all 40 enrolled BWL-AI participants completed a 5-item self-report survey. The first item asked participants to rate the extent to which the coaching messages had helped them to lose weight overall. The remaining items asked participants to rate how well the coaching messages had fulfilled a series of additional objectives, including (1) understanding progress toward their goals, (2) making the changes to their behavior required to lose weight, (3) implementing new weight loss strategies, and (4) staying motivated in the face of challenges. Ratings were obtained on a 1-5 Likert scale where 5 represents a more favorable rating (1=“not at all”; 5=“extremely”).

###### Qualitative Assessment

In addition to the self-report survey, all participants attended a 15- to 30-minute individual structured videoconference interview conducted by a member of the Project ReLearn study staff, all of whom had a bachelor’s degree or higher and a background in health psychology. The interview was completed for 39 out of the 40 participants completing the acceptability survey (for 1 participant, the interview was not completed due to scheduling difficulties). Participants were informed that the purpose of the interview was to gather feedback on the coaching messages so as to help improve these messages in the future.

Open-ended feedback on the messaging system was elicited using 5 scripted questions. Participants’ responses to each question were transcribed verbatim and in real time by the assessor. (These transcripts were not returned to participants, who did not provide feedback on the findings.) Assessors were instructed not to ask any additional probing questions. Participants were first asked: “How have you felt overall about the coaching messages?” Then, participants were shown (via screen-share) the most recent coaching message that they had received during a week in which they had lost weight. While this message was displayed, participants were asked:

Sometimes, you received coaching messages on weeks when you lost weight. Here is an example. During the weeks when you lost weight, how helpful did you find the coaching messages for keeping you on track and motivated during the following week?

As it was anticipated that participants would have different reactions to messages received on weeks where weight was lost versus gained, participants were then shown the most recent message that they had received during a week when they had either gained or maintained weight and asked:

Sometimes, you received coaching messages on weeks when you gained or maintained your weight. Here is an example. During weeks such as these, how helpful did you find the coaching messages for keeping you on track and motivated during the following week?

Participants were then asked the following, while still viewing the second message: “Looking at this same message again now, is there anything that you wish were included that isn't here?” Finally, participants were asked: “Overall, what could be better about the coaching messages?”

##### Data Analysis

Responses to self-report survey items were summarized using descriptive statistics. To synthesize feedback from the structured interviews, we used the Braun and Clarke [33] thematic analysis method. In brief, thematic analysis is an inductive data analytic approach in which 2 or more reviewers each independently review a qualitative data set and assign interpretive “codes” to each inferred unit of meaning. These codes are then organized into higher level “themes,” ensuring that every code is linked to at least 1 theme. Two coders (MB and LT), both clinical psychology doctoral students (1 master’s degree level and 1 bachelor’s degree level) and 1 (LT) who had prior formal training in thematic analysis, analyzed the data from the structured interviews. Both coders read the full set of interview transcripts, each independently generating a set of initial codes and preliminary themes. The 2 sets of results were then combined into a single merged list of codes, which is organized by theme. This list was then condensed and restructured through a series of collaborative face-to-face discussions between the 2 coders until a final consensus was reached on how to organize codes within themes and themes within meta-themes. Disagreements between coders were resolved both through these discussions and the exchange of written drafts between coders.

## Results

### Participant Characteristics

The average age across the participant sample at study enrollment (N=40) was 50.23 (SD 11.01; range 30-69) years, with a mean BMI of 35.81 (SD 6.07) kg/m^2^. Self-reported racial identity was Asian (n=2, 5%), Black (n=6, 15%), Hawaiian or Pacific Islander (n=1, 2.5%), White (n=32, 80%), more than 1 race (n=2, 5%), and not reported (n=1, 2.5%); participants could select more than 1 option, and thus the sum of percentages exceeds 100%. Overall, 7.5% (n=3) of the sample reported Latino or Latina or Hispanic identity. Self-reported gender identity was female (n=34, 85%) and male (n=6, 15%). In total, 67.5% (n=27) of participants reported current full-time employment. In addition, 22.5% (n=9) reported not currently working outside the home (eg, retired), 5% (n=2) reported part-time employment, and 5% (n=2) reported receiving disability or Supplemental Security Income. The mean self-reported annual salary was US $82,495 (SD US $46,929) across the sample; however, only 25 out of 40 participants opted to provide this information. The mean percent weight loss from baseline at the time of assessment was 6.7% (SD 4.48%) for participants assessed at 6 months and 9.69% (SD 7.51%) for participants assessed at 12 months (n=40 for both time points)

### Acceptability Measures

Descriptive statistics for acceptability items are presented in Table 1. Mean overall acceptability was moderate (2.67, SD 1.27), with 60% (n=24) of participants rating the messages as “somewhat helpful” or greater. Ratings were highest for the messages’ ability to help participants understand their progress toward goals and lowest for the messages’ ability to help participants implement new strategies for weight loss.

**Table 1:** Descriptive statistics for acceptability measures (n=40). All items are on a 1-5 Likert scale where higher scores indicate higher acceptability.

Acceptability measure	Value, mean (SD)	Value, median (IQR)	Rating 3 or higher, n (%)	Skewness
Overall helpfulness for weight loss	2.67 (1.27)	3 (2-4)	24 (60)	0.008
Understand how I was progressing toward my goals	3.28 (1.45)	3 (3-4)	30 (75)	–0.35
Make changes to behavior to lose weight	2.58 (1.32)	3 (1-3)	22 (55)	0.33
Implement new strategies	2.38 (1.31)	2 (1-3)	19 (48)	0.46
Stay motivated in the face of barriers	2.88 (1.44)	3 (2-4)	24 (60)	0.12

### Thematic Analysis Results

#### Overview

A summary of meta-themes and subthemes alongside representative quotes is presented in Table S2 in Multimedia Appendix 1. Two distinct meta-themes emerged from the thematic analysis of interview transcripts: (1) messages were effective, low-commitment weekly reminders of intervention goals and skills and (2) participants wanted a messaging system that could “get to know” them personally and understand their lived experiences with weight loss. Within these 2 meta-themes, 12 subthemes were identified (each accompanied by representative quotes) as described in the sections that follow. For each theme, the total number of supporting codes, summed across coders, is presented as an approximate measure of prevalence within the data set. Due to the similarity of feedback obtained between assessment points (ie, 6 vs 12 months), themes were collapsed across time points during the thematic analysis rather than evaluated separately.

#### Meta-Theme 1, Subtheme 1: Weekly Synthesis of Personal Data Is Helpful (26 Codes)

Several participants praised the summary box included at the beginning of each coaching message, noting that it provided a useful objective measure of goal progress. One participant also noted that the summary box helped with identifying specific areas of adherence that could be improved:

I think [the messages] were good. They really stated “hey, here's some things that we can see that you aren't doing”. They were kindly worded and still had positive reinforcement. I really liked them. I'm a visual learner, so seeing things like that is a lot easier.

Participant #16

#### Meta-Theme 1, Subtheme 2: Messages Are Most Helpful When Succinct and Easy to Understand (21 Codes)

A few participants commented that they found the messages to be most useful when they are succinct and do not require much time to read. One participant specifically reported being pleased when they were assigned to receive messages due to the low time commitment involved: “I loved the coaching messages because they could be consumed on my timeline. It was an easily digestible piece of data and actionable” [Participant #10]. Conversely, a few participants criticized examples of messages they received that they found overly lengthy. Several participants also provided suggestions for how the messages could be made more concise or easier to quickly process (eg, including subheadings for each messaging component).

#### Meta-Theme 1, Subtheme 3: Messages Provide Useful Reminders of Goals and Skills (19 Codes)

One positive aspect of the coaching messages noted by participants was that they are useful for keeping program goals (eg, target calorie range) and behavioral skills (eg, dietary self-monitoring) in mind, given that messages are delivered on weeks without any counselor contact. For example:

[The messages] definitely highlighted how important tracking was, and that's something I had to learn. I was a terrible tracker, and I tried to track before on my own... So, [the messages] are a great reminder to track and it’s so true.

Participant #18

#### Meta-Theme 1, Subtheme 4: Messages Provide Goal Accountability (11 Codes)

Several participants noted that the coaching messages helped to sustain perceptions that their data are actively monitored. For example:

If I'm looking at the app or Fitbit, I can't tell how much I'm losing just by that. It's good to know that someone is behind the scenes watching and knows what I'm doing.

Participant #37

Another participant suggested that the messages served as a useful reminder that the program had not paused, just because no counselor contact occurred in the past week: “[The message] is giving me knowledge that you guys are checking my logs, and you are still there” [Participant #22].

#### Meta-Theme 2, Subtheme 1: Participants Want Messages to be Validating and Encouraging, Regardless of Goal Attainment (57 Codes)

Some participants reported wishing that the coaching messages avoided drawing attention to poor progress, and instead provided more validation and encouragement in response to challenges. For example:

I would prefer if the message could be a little more positive instead of nagging me just to do better. Instead, maybe it should tell the person “You've got this” and then ask me to create a specific action plan for the next week.

Participant #40

Other participants reported that coaching messages came across as critical without always offering helpful suggestions for improvement, which could feel demoralizing, especially during weeks when they felt that they had given their best effort (“The truth is there were weeks I did [everything], and I still didn't lose weight because our bodies just are like that” [Participant #5]).

#### Meta-Theme 2, Subtheme 2: Messages Seem to Lack Empathy, Which Causes Them to Feel Robotic or Impersonal (48 Codes)

A common critique of the coaching messages was that they did not reflect understanding or empathy surrounding the challenges that participants experienced while losing weight. One participant noted:

The coaching messages could not feel so impersonal and so automated. As they exist right now, they don't speak to me in any way. They're frustrating. They don't connect to what my issue or my success is.

Participant #39

Several participants also reported wishing that coaching messages could understand and tailor feedback based on unexpected life circumstances (eg, personal losses) that had temporarily made weight loss more challenging. Other participants criticized the messages for being over-reliant on data summaries without providing enough empathy, noting that this could come across as invalidating or demeaning (eg, “[This message] re-stating multiple times that you gained 4.4 pounds is a slap in the face” [Participant #31]).

#### Meta-Theme 2, Subtheme 3: Messages Are not Sufficiently Personalized (42 Codes)

Many participants reported wishing that the coaching messages could know more about them as individuals generally. Participants referenced shortcomings in this area such as the lack of referring to participants by their names, not understanding dietary needs or preferences (eg, dietary restrictions), and not understanding participants’ typical dietary patterns outside of weekly calorie intake averages.

#### Meta-Theme 2, Subtheme 4: Messages Lack Salience or Feel Unimportant (32 Codes)

Several participants indicated that the coaching messages generally had little impact on their day-to-day weight loss efforts, especially compared to meetings with a human counselor. For example:

For me, I read [the message] in that amount of time you had it on the screen, then it's over, I'm onto the next thing in my life. If I was sitting in a 15-minute call, we'd talk about it longer, or in the hour group, we really talk about it.

Participant #18

Other participants stated that because they knew the messages were written by a computer system, and not a human, this made them feel less relevant or important (eg, “I don’t need a machine to tell me what to do” [Participant #9]).

#### Meta-Theme 2, Subtheme 5: Messages Sometimes Overlook Important Data Patterns (32 Codes)

Many participants found that the coaching messages did not always provide feedback on the domains that would have been most useful. For example, a few participants reported that the coaching messages sometimes overemphasized relatively small changes in adherence in 1 domain (eg, a very small increase in physical activity from the previous week) without addressing more meaningful changes in other domains (eg, a large increase in calorie intake). Several participants also reported that coaching messages underemphasized *overall* levels of adherence while overemphasizing *relative* changes in adherence (eg, “[The message] says I'm on an upward trajectory with my food tracking, but it's still short of optimal. That should have been woven into the message” [Participant #10]). Other participants expressed a desire for the coaching messages to provide more fine-grained summaries of data (eg, on food types consumed or on how adherence varied from day to day).

#### Meta-Theme 2, Subtheme 6: Messages Often Restate Information That Participants Already Know (31 Codes)

Another critique of the coaching messages was that they offered little information that participants did not already know, causing the messages to feel redundant or lacking in usefulness. For example:

*Iunderstand this is one of the tools, but it's not motivating me. It's not telling me anything I wouldn't consider myself... generally speaking, on the weeks I gained weight, I was aware. I knew that that was happening*. [Participant #39]

#### Meta-Theme 2, Subtheme 7: Feedback From Messages Can be Confusing or Misaligned With Participants’ Expectations (20 Codes)

A few participants expressed concerns about the data summaries within the coaching messages being misaligned with their personal impressions of their goal progress (eg, “I think [the messages] don’t necessarily line up with the weekly statistics” [Participant #33]). Relatedly, a few participants suggested that coaching messages should provide date and time ranges for each data summary to help resolve discrepancies between messaging content and participants’ own perceptions.

#### Meta-Theme 2, Subtheme 8: Messages Should Be Interactive (6 Codes)

Finally, a few participants wished that the “messaging” tab of the ReLearn app could be used to interact directly with coaches (eg, to ask a question about the feedback they received through the coaching messages). Other participants indicated that they would have liked for the coaching messages to be more conversational or for the messaging system to allow for 2-way communication (eg, “It would be more helpful if there were the ability to respond, or if there were a question asked with an expected response...” [Participant #40]).

## Discussion

Automated messaging is a widely used intervention component in mHealth weight loss treatments [10,13,16,17], but its efficacy is limited [10,19]. Few studies have explored user experiences with more sophisticated automated messaging–based interventions [12,23-25], which have become more widely used during the past half decade [17,21,34], impeding efforts to enhance the effectiveness of this intervention modality for weight loss. Thus, in this study, we evaluated the experiences of 40 individuals with overweight or obesity who received relatively sophisticated (highly individually tailored and data-driven) automated messages as part of a year-long behavioral weight loss treatment program. The automated messaging system provided participants with comprehensive, individually tailored weekly feedback on progress toward weight and behavioral goals each week. The central aim of this report was to synthesize qualitative feedback obtained on the strengths and weaknesses of the automated messaging system through structured interviews. For additional context, we also detailed the design of the messaging system and presented quantitative outcome data related to messaging acceptability across 5 domains.

Overall, participants indicated only moderate levels of satisfaction with the helpfulness of the system, providing a mean rating of 2.67 (SD 1.27) on a 5-point Likert scale. The proportion of participants finding the messaging system to be at least “somewhat helpful” (24/40, 60%) was somewhat lower in this study compared to prior studies evaluating the acceptability of messaging systems for weight loss [23,35-37]. This discrepancy may be due to Project ReLearn participants having higher expectations for the system’s capabilities compared to participants in prior qualitative studies, due to the proliferation of highly sophisticated automated messaging technology (eg, conversational AI) over the past several years. Another potential reason could be that participants in this study received automated messages alongside human counselor support within the behavioral weight loss program, leading some to unfavorably compare the coaching messages to face-to-face counseling. As such, compared to prior qualitative studies, the estimates of messaging acceptability provided in this report could provide a more accurate reflection of weight loss–seeking individuals’ perceptions of automated messaging when referenced against more standard human-delivered counseling.

Across the acceptability domains examined, participants in this study found the automated messages to be most useful for helping them understand their progress toward their goals (30/40, 75% provided a rating of “somewhat helpful” or higher in this domain). This finding is perhaps expected, given the strong emphasis of the coaching messages on detailed data summaries (which as previously noted, has been lacking in many prior messaging systems). During the structured interviews, participants highlighted several additional aspects of the coaching messages that they found useful. In particular, many indicated that the messages provided effective, low-commitment weekly reminders of intervention goals and skills (meta-theme 1). This feedback generally aligns with prior studies evaluating messaging systems for weight loss [12,23,24] and underscores the usefulness of automated messages as a tool to maintain goal or skill salience (eg, reminding individuals of strategies they can use to stay within daily calorie targets) in the absence of human counselor intervention.

Participants in this study found the coaching messages least helpful for suggesting new behavioral weight loss strategies, out of the 5 acceptability domains examined. One prior study similarly reported that a text messaging intervention for weight loss was rated as less useful for suggesting new behavioral strategies than it was for providing motivational support [38]. Perhaps, these low ratings reflect that the targeted strategy suggestions included in the messages were not as well-explained or were lacking in detail relative to suggestions provided by their human weight loss counselors. During the structured interviews, participants also indicated that these strategy suggestions often felt insufficiently tailored (eg, were not feasible due to a participant’s dietary restrictions or current life circumstances). Thus, our findings draw attention to the importance of providing users automated messages for weight loss with detailed, tailored, and context-sensitive behavioral strategy suggestions. It is also possible that automated messaging as an intervention modality is limited to promoting the acquisition of new skills, which may require more intensive human counselor support than promoting the maintenance of existing skills.

During the structured interviews, participants also expressed a desire for the automated messages to better get to know them and their lived experiences with weight loss (meta-theme 2). A notable emergent theme within this meta-theme was that many participants found the messages to lack adequate empathy or encouragement in response to challenges. Other themes included that messages felt overly generic, vague, or irrelevant to participants’ current needs and that the messages should have been more interactive and allowed for 2-way communication, as has been implemented in some prior automated messaging systems [16,17]. Broadly, participants’ suggestions for improving the automated messaging system in Project ReLearn align with prior reports that have identified messaging personalization or tailoring [24,25,35,38] and emotional encouragement [24,38] as important for maintaining engagement with automated messaging over time. Participants’ critiques that the messages did not understand them on an individual level (ie, meta-theme 2) also highlight a need for future systems capable of tailoring messaging feedback based on variables other than weight and intervention adherence. For example, messages could additionally be tailored based on users’ self-reported emotional states or situational factors impacting adherence (eg, acknowledging that a participant may need to relax some of their intervention goals while traveling, but encouraging continued adherence to certain core skills such as dietary self-monitoring).

One limitation of this study is that data collected via structured interviews were not audio recorded and were instead transcribed in real time by an assessor, which could have potentially caused inaccuracies or bias. Participants in this sample also reported a higher income level (and thus potentially higher socioeconomic status) compared to the US average [39], potentially affecting the generalizability of the results. Another limitation is that at the time of data collection, participants had been receiving the automated messages for somewhat different lengths of time (ie, 6 vs 12 months). However, we contend that our findings still have importance for refining future automated messaging systems for weight loss and note that qualitative findings appeared highly similar between assessment points. For example, our results highlight the importance of tailoring messaging content to users’ individual preferences and evolving life circumstances. As such, we recommend that researchers designing future automated messaging systems consider allowing users to provide direct input into the content or tone of the messages they receive. For example, some prior systems have allowed users to craft their own coaching messages [37] or tailored messages based on users’ self-reported behavior change goals [17] or self-reported current stage of change [40]. Relatedly, we also recommend that future systems be conversational (ie, allow for ongoing 2-way communication between users and the system), to further increase users’ degree of control over the messaging content they receive and create a more individually tailored experience.

As previously noted, the emergence of conversational AI (eg, chatbots) that can engage interactively with individuals and adopt a conversational tone [20] may help to address some of the concerns noted above. Compared to the messaging system used in this study, conversational AI is presumably much better able to adapt messaging tone and content to users’ specific preferences, provide validation, and prompt user self-reflection. Nonetheless, future comparative trials will be required to establish whether conversational AI systems are in fact more acceptable to weight loss–seeking individuals than traditional messaging systems lacking conversational capabilities, such as the system used in this study. Furthermore, increased messaging acceptability may not translate to increased weight loss efficacy. For example, a possible advantage of the automated messaging system that we evaluated versus conversational AI is the higher degree of domain-specific (ie, weight loss) expertise, given that all messaging content was crafted and curated by a team of health psychology experts. As such, it will also be important to evaluate the comparative weight loss efficacy of different methods for constructing automated messaging–based interventions for weight loss. Hopefully, continued advances within automated messaging technology, as well as mHealth for weight control more broadly, will help to curb the global epidemic of obesity.

## Appendix

Multimedia Appendix 1 Overview of behavioral feedback message themes, and emergent themes and subthemes from the thematic analysis of qualitative message acceptability feedback and representative quotes.

## Abbreviations

AI: artificial intelligence
BWL-AI: AI-optimized behavioral weight loss group
BWL-S: standard behavioral weight loss group
mHealth: mobile health
MVPA: moderate to vigorous activity

## Data Availability

The data sets generated during and analyzed during this study are available in the Open Science Framework digital repository [41].

## References

[1] (2023). Obesity and overweight. World Health Organization.

[2] (2023). Overweight and obesity statistics. National Institute of Diabetes and Digestive and Kidney Diseases.

[3] Abdelaal M, le Roux CW, Docherty NG (2017). Morbidity and mortality associated with obesity. Ann Transl Med.

[4] Wadden TA, Tronieri JS, Butryn ML (2020). Lifestyle modification approaches for the treatment of obesity in adults. Am Psychol.

[5] Williamson DA, Bray GA, Ryan DH (2015). Is 5% weight loss a satisfactory criterion to define clinically significant weight loss?. Obesity (Silver Spring).

[6] Jensen P, Zachariae C, Christensen R, Geiker NRW, Schaadt BK, Stender S, Astrup A, Hansen PR, Skov L (2014). Effect of weight loss on the cardiovascular risk profile of obese patients with psoriasis. Acta Derm Venereol.

[7] Janssen EM, Jerome GJ, Dalcin AT, Gennusa JV, Goldsholl S, Frick KD, Wang NY, Appel LJ, Daumit GL (2017). A cost analysis of implementing a behavioral weight loss intervention in community mental health settings: results from the ACHIEVE trial. Obesity (Silver Spring).

[8] Krukowski RA, Tilford JM, Harvey-Berino J, West DS (2011). Comparing behavioral weight loss modalities: incremental cost-effectiveness of an internet-based versus an in-person condition. Obesity (Silver Spring).

[9] Cavero-Redondo I, Martinez-Vizcaino V, Fernandez-Rodriguez R, Saz-Lara A, Pascual-Morena C, Álvarez-Bueno C (2020). Effect of behavioral weight management interventions using lifestyle mHealth self-monitoring on weight loss: a systematic review and meta-analysis. Nutrients.

[10] Skinner R, Gonet V, Currie S, Hoddinott P, Dombrowski SU (2020). A systematic review with meta-analyses of text message-delivered behaviour change interventions for weight loss and weight loss maintenance. Obes Rev.

[11] Berrouiguet S, Baca-García E, Brandt S, Walter M, Courtet P (2016). Fundamentals for future mobile-Health (mHealth): a systematic review of mobile phone and web-based text messaging in mental health. J Med Internet Res.

[12] Shaw RJ, Bosworth HB, Hess JC, Silva SG, Lipkus IM, Davis LL, Johnson CM (2013). Development of a theoretically driven mHealth text messaging application for sustaining recent weight loss. JMIR Mhealth Uhealth.

[13] Ahn A, Choi J (2016). A one-way text messaging intervention for obesity. J Telemed Telecare.

[14] Shapiro JR, Koro T, Doran N, Thompson S, Sallis JF, Calfas K, Patrick K (2012). Text4Diet: a randomized controlled study using text messaging for weight loss behaviors. Prev Med.

[15] Haapala I, Barengo NC, Biggs S, Surakka L, Manninen P (2009). Weight loss by mobile phone: a 1-year effectiveness study. Public Health Nutr.

[16] Godino JG, Golaszewski NM, Norman GJ, Rock CL, Griswold WG, Arredondo E, Marshall S, Kolodziejczyk J, Dillon L, Raab F, Jain S, Crawford M, Merchant G, Patrick K (2019). Text messaging and brief phone calls for weight loss in overweight and obese English- and Spanish-speaking adults: a 1-year, parallel-group, randomized controlled trial. PLoS Med.

[17] Lin M, Mahmooth Z, Dedhia N, Frutchey R, Mercado CE, Epstein DH, Preston KL, Gibbons MC, Bowie JV, Labrique AB, Cheskin LJ (2015). Tailored, interactive text messages for enhancing weight loss among African American adults: the TRIMM randomized controlled trial. Am J Med.

[18] Berry MP, Chwyl C, Metzler AL, Sun JH, Dart H, Forman EM (2022). Associations between behaviour change technique clusters and weight loss outcomes of automated digital interventions: a systematic review and meta-regression. Health Psychol Rev.

[19] Job JR, Fjeldsoe BS, Eakin EG, Reeves MM (2018). Effectiveness of extended contact interventions for weight management delivered via text messaging: a systematic review and meta-analysis. Obes Rev.

[20] Chew HSJ (2022). The use of artificial intelligence-based conversational agents (Chatbots) for weight loss: scoping review and practical recommendations. JMIR Med Inform.

[21] Stein N, Brooks K (2017). A fully automated conversational artificial intelligence for weight loss: longitudinal observational study among overweight and obese adults. JMIR Diabetes.

[22] Eborall H, Morton K, Morgan DL, Barbour RS (2017). Use of focus groups in developing behavioural mHealth interventions: a critical review. A New Era in Focus Group Research: Challenges, Innovation and Practice.

[23] Spark LC, Fjeldsoe BS, Eakin EG, Reeves MM (2015). Efficacy of a text message-delivered extended contact intervention on maintenance of weight loss, physical activity, and dietary behavior change. JMIR Mhealth Uhealth.

[24] Job JR, Spark LC, Fjeldsoe BS, Eakin EG, Reeves MM (2017). Women's perceptions of participation in an extended contact text message-based weight loss intervention: an explorative study. JMIR Mhealth Uhealth.

[25] Smith KL, Kerr DA, Fenner AA, Straker LM (2014). Adolescents just do not know what they want: a qualitative study to describe obese adolescents' experiences of text messaging to support behavior change maintenance post intervention. J Med Internet Res.

[26] Forman EM, Berry MP, Butryn ML, Hagerman CJ, Huang Z, Juarascio AS, LaFata EM, Ontañón S, Tilford JM, Zhang F (2023). Using artificial intelligence to optimize delivery of weight loss treatment: protocol for an efficacy and cost-effectiveness trial. Contemp Clin Trials.

[27] Ryan K, Dockray S, Linehan C (2019). A systematic review of tailored eHealth interventions for weight loss. Digit Health.

[28] Amagai S, Pila S, Kaat AJ, Nowinski CJ, Gershon RC (2022). Challenges in participant engagement and retention using mobile health apps: literature review. J Med Internet Res.

[29] Teeriniemi AM, Salonurmi T, Jokelainen T, Vähänikkilä H, Alahäivälä T, Karppinen P, Enwald H, Huotari ML, Laitinen J, Oinas-Kukkonen H, Savolainen MJ (2018). A randomized clinical trial of the effectiveness of a web-based health behaviour change support system and group lifestyle counselling on body weight loss in overweight and obese subjects: 2-year outcomes. J Intern Med.

[30] Napolitano MA, Hayes S, Bennett GG, Ives AK, Foster GD (2013). Using Facebook and text messaging to deliver a weight loss program to college students. Obesity (Silver Spring).

[31] Trella AL, Zhang KW, Nahum-Shani I, Shetty V, Doshi-Velez F, Murphy SA (2022). Designing reinforcement learning algorithms for digital interventions: pre-implementation guidelines. Algorithms.

[32] Sutton RS, Barto AG (2018). Reinforcement Learning: An Introduction, Second Edition.

[33] Braun V, Clarke V (2006). Using thematic analysis in psychology. Qual Res Psychol.

[34] Everett E, Kane B, Yoo A, Dobs A, Mathioudakis N (2018). A novel approach for fully automated, personalized health coaching for adults with prediabetes: pilot clinical trial. J Med Internet Res.

[35] Jensen CD, Duraccio KM, Barnett KA, Fortuna C, Woolford SJ, Giraud-Carrier CG (2019). Feasibility, acceptability, and preliminary effectiveness of an adaptive text messaging intervention for adolescent weight control in primary care. Clin Pract Pediatr Psychol.

[36] Chow CK, Redfern J, Hillis GS, Thakkar J, Santo K, Hackett ML, Jan S, Graves N, de Keizer L, Barry T, Bompoint S, Stepien S, Whittaker R, Rodgers A, Thiagalingam A (2015). Effect of lifestyle-focused text messaging on risk factor modification in patients with coronary heart disease: a randomized clinical trial. JAMA.

[37] Gerber BS, Stolley MR, Thompson AL, Sharp LK, Fitzgibbon ML (2009). Mobile phone text messaging to promote healthy behaviors and weight loss maintenance: a feasibility study. Health Informatics J.

[38] Mhurchu CN, Whittaker R, McRobbie H, Ball K, Crawford D, Michie J, Jiang Y, Maddison R, Waterlander W, Myers K (2014). Feasibility, acceptability and potential effectiveness of a mobile health (mHealth) weight management programme for New Zealand adults. BMC Obes.

[39] (2022). Income in the United States: 2021. United States Census Bureau.

[40] Partridge SR, McGeechan K, Hebden L, Balestracci K, Wong AT, Denney-Wilson E, Harris MF, Phongsavan P, Bauman A, Allman-Farinelli M (2015). Effectiveness of a mHealth lifestyle program with telephone support (TXT2BFiT) to prevent unhealthy weight gain in young adults: randomized controlled trial. JMIR Mhealth Uhealth.

[41] Berry M (2023). User experiences with an automated messaging intervention delivered synchronously with behavioral weight loss treatment. OSF.

