# Supplementary material for: Automated Messaging Delivered Alongside Behavioral Treatment for Weight Loss: Qualitative Study
Source: JMIR Form Res. 2023 Nov 6;7:e50872. doi: 10.2196/50872 (PMC10660236; doi:10.2196/50872)
Supplement: Multimedia Appendix 1 [file formative_v7i1e50872_app1.docx]

**Multimedia Appendix**

**Table S1.** Overview of behavioral feedback message themes, with examples of “parent” messages, and probabilities of receiving each theme for an example participant who has gained weight since starting the program and consistently gained weight during the past month.

| Message Theme | Example Parent Message | Probability of selection |
| --- | --- | --- |
|  |  |  |
| Struggling with Goal | It looks like physical activity has been especially tough this week. Maybe you could try [strategy] to help increase your activity for next week? | 15% |
| Goal Improving | I noticed that you were able to track your food EVERY day this week - that's fantastic! | 40% |
| Goal Declining | Your calories this week were higher than your average over the past month; it seems like this might be part of why your weight increased this week. Getting a handle on this so it doesn't become trend is really important! To get back on track, try [strategy] | 0% |
| Goal Maintenance | Whoa, it looks like you tracked your calories really consistently this month! | 10% |
| General Motivation | Great work staying engaged with the program! Behavior change is a long process, so learning the skills and keeping up with the program sets a good foundation for the long term! | 35% |

**Table S2.** Emergent themes and subthemes from the thematic analysis of qualitative message acceptability feedback and representative quotes.

| **Emergent themes and subthemes** | **Representative Quotes** |
| --- | --- |
|  | |
| **Meta-theme 1: Messages are effective, low-commitment weekly reminders of intervention goals and skills** | |
|  |  |
| Subtheme 1: Weekly synthesis of personal data is helpful | *“It gave me a concrete piece of information to work with over the next week.”*    *“I think [the messages] were good. They really stated "hey, here's some things that we can see that you aren't doing". They were kindly worded and still had positive reinforcement. I really liked them. I'm a visual learner, so seeing things like that is a lot easier.”* |
| Subtheme 2: Messages are most helpful when succinct and easy to understand | *“For myself, it needs to be easy to read and digest and very clear and concise. And I thought some of those little paragraphs [in this message] got too wordy and verbose.”*    *“I loved the coaching messages because they could be consumed on my timeline. It was an easily digestible piece of data and actionable.”*    *Sometimes it can feel like a lot to keep on top of the messages... I think it would be nice if each individual message had a header or title (e.g., weight feedback) to better organize things.”* |
| Subtheme 3: Messages provide useful reminders of goals and skills | *“[The messages] definitely highlighted how important tracking was, and that's something I had to learn. I was a terrible tracker, and I tried to track before on my own, using Weight Watchers’ point tracking, which is even more simple than this, and I never stuck to it. So, [the messages] are a great reminder to track and it’s so true. You almost had to beat me over the head with the idea of tracking.”* |
| Subtheme 4: Messages provide goal accountability | *“If I'm looking at the app or Fitbit, I can't tell how much I'm losing just by that. It's good to know that someone is behind the scenes watching and knows what I'm doing.”*    *“[The message] is giving me knowledge that you guys are checking my logs, and you are still there. You know what I am doing, or lack thereof.”* |
| **Meta-Theme 2: Participants want a messaging system that can “get to know” them personally and understand their lived experiences with weight loss.** | |
| Subtheme 1: Participants want messages to be validating and encouraging, regardless of goal attainment | *“I would prefer if the message could be a little more positive instead of nagging me just to do better. Instead, maybe it should tell the person ‘you've got this’ and then ask me to create a specific action plan for the next week.”*    *“[The message] is really only looking at three things. Did you track, and if you did, where is it, did you exercise, and did you eat in the calorie range. The truth is there were weeks I did all those things, and I still didn't lose weight because our bodies just are like that.”*    *“[The message] just says to try to identify what might have gone differently — I found that insulting.”* |
| Subtheme 2: Messages seem to lack empathy, which causes them to feel robotic or impersonal | *“The coaching messages could not feel so impersonal and so automated. As they exist right now, they don't speak to me in any way. They're frustrating. They don't connect to what my issue or my success is.”*    *“[This message came] the week I was planning my Mom’s funeral and could have cared less. The message doesn't know me. I know that [clinician names] and the people in my program knew that my Mom died--and they were very sweet to me. But that message still comes in, and I ignored it.”*    *“The coaching messages I've received — maybe because it's me because I haven't put in any data or haven't logged any food — it feels like whatever... I'm looking at my weekly summary and going back to December. Everything's at 0. It sends me a message saying that we don't have enough data on your weight to tell how much you've lost this week... All of the data is 0. Is anyone looking at this really? Does anybody care?”*    *“[This message] restating multiple times that you gained 4.4 pounds is a slap in the face. [It should] just say you had a gain this week, rather than reiterating it. There might be a softer way to say it... You just have the data, just eliminate the first sentence, rather than having three times the same “you gained weight, you gained weight, you gained weight".* |
| Subtheme 3: Messages are not sufficiently personalized | *“[I’d like] more specific suggestions... like, if someone was eating lots of ice-cream– you could [take] a deeper dive into what I ate that past week.”* |
| Subtheme 4: Messages lack salience or feel unimportant | *“For me, I read [the message] in that amount of time you had it on the screen, then it's over, I'm onto the next thing in my life. If I was sitting in a 15-minute call, we'd talk about it longer, or in the hour group, we really talk about it.”*    *“It’s AI — no one's looking at you and making assessment based on what you did.”* |
| Subtheme 5: Messages sometimes overlook important data patterns | *“[During this week] I was in the calorie range and tracked my food just one day. I haven't been good about tracking my food. Because I went from 0 to 1, [the message] says it’s an upward trajectory. I don't feel like that's a valid encouragement. There wasn't a substance to the message received.”*    *“[The message] says I'm on an upward trajectory with my food tracking, but it's still short of optimal. That should have been woven into the message.”* |
| Subtheme 6: Messages often restate information that participants already know | *“I understand this is one of the tools, but it's not motivating me. It's not telling me anything I wouldn't consider myself... generally speaking on the weeks I gained weight, I was aware. I knew that that was happening.”* |
| Subtheme 7: Feedback from messages can be confusing or misaligned with participants’ expectations | *“I think that they don't necessarily line up with the weekly statistics.”*    *“It feels like I’m getting cheerleading for not doing what I should have done.”* |
| Subtheme 8: Messages should be interactive | *“It would be more helpful if there were the ability to respond, or if there were a question asked with an expected response... For example, maybe it could ask me "What are the steps you will take to change things up next week?" And then you could reply with your answer.”* |
